# Detecting Selection in the Blue Crab, *Callinectes sapidus*, Using DNA Sequence Data from Multiple Nuclear Protein-Coding Genes

**DOI:** 10.1371/journal.pone.0099081

**Published:** 2014-06-04

**Authors:** Bree K. Yednock, Joseph E. Neigel

**Affiliations:** Biology Department, University of Louisiana at Lafayette, Lafayette, Louisiana, United States of America; University of Louisiana at Lafayette, United States of America

## Abstract

The identification of genes involved in the adaptive evolution of non-model organisms with uncharacterized genomes constitutes a major challenge. This study employed a rigorous and targeted candidate gene approach to test for positive selection on protein-coding genes of the blue crab, *Callinectes sapidus*. Four genes with putative roles in physiological adaptation to environmental stress were chosen as candidates. A fifth gene not expected to play a role in environmental adaptation was used as a control. Large samples (n>800) of DNA sequences from *C. sapidus* were used in tests of selective neutrality based on sequence polymorphisms. In combination with these, sequences from the congener *C. similis* were used in neutrality tests based on interspecific divergence. In multiple tests, significant departures from neutral expectations and indicative of positive selection were found for the candidate gene trehalose 6-phosphate synthase (*tps*). These departures could not be explained by any of the historical population expansion or bottleneck scenarios that were evaluated in coalescent simulations. Evidence was also found for balancing selection at ATP-synthase subunit 9 (*atps*) using a maximum likelihood version of the Hudson, Kreitmen, and Aguadé test, and positive selection favoring amino acid replacements within ATP/ADP translocase (*ant*) was detected using the McDonald-Kreitman test. In contrast, test statistics for the control gene, ribosomal protein L12 (*rpl*), which presumably has experienced the same demographic effects as the candidate loci, were not significantly different from neutral expectations and could readily be explained by demographic effects. Together, these findings demonstrate the utility of the candidate gene approach for investigating adaptation at the molecular level in a marine invertebrate for which extensive genomic resources are not available.

## Introduction

According to the neutral theory of molecular evolution, natural selection acts mainly to remove deleterious mutations from populations, leaving behind allelic variation that is almost entirely neutral [1], [2]. The enduring value of this theory is that it predicts how genetic variation should be distributed within and between populations by non-selective forces such as gene flow and genetic drift. Consequently, the neutral theory has had a lasting influence in population genetics, molecular phylogenetics, and conservation biology. However, as the genomes of more species have been sequenced, it has become evident that natural selection acts on variation at a greater proportion of loci than anticipated by the classical neutral theory. Evidence for widespread selection has been found in the genomes of model organisms including *Drosophila* [3]–[5], *Arabidopsis* [6], [7], and *Homo sapiens* [8]–[10]. Despite fewer genomic resources, studies of non-model organisms have also provided evidence for the importance of natural selection at the molecular level [11]–[14].

Since its introduction, the neutral theory has provided null models for statistical tests designed to detect selection [15], [16]. These include tests based on sequence polymorphism within a single population (although typically the entire species is considered the population) [17], [18], comparison of homologous sequences between species [16], detection of outlier loci with anomalously high or low levels of divergence between populations [19], and correlations between alleles and environmental variables [20]. Although most of these tests use selective neutrality as a null hypothesis, they are most valuable when they can also distinguish between purifying selection, which is expected to be acting on most genes, and diversifying, balancing, or positive selection.

One approach that is increasingly used to find outlier loci in non-model organisms is the genome-wide scan for single nucleotide polymorphisms (SNPs) experiencing positive selection [21]. Genome scans of marine fish have revealed a number of candidate SNPs [22], [23]. However, in a non-model organism for which a reference genome is not available, the genomic locations of SNPs are often unknown, leaving the identity and adaptive significance of loci that appear to be under selection unknown as well. An alternative approach is to sequence candidate genes that have functions that suggest *a priori* they could be subject to positive selection. Using DNA sequences rather than SNPs has the added advantage of avoiding ascertainment bias, which is the tendency to overlook polymorphisms that are absent or occur in very low frequencies in the small groups of individuals that are screened during the initial SNP discovery process [24]. DNA sequencing also reveals all of the variation in the targeted region, is highly reproducible, and permits the use of well-developed sequence-based neutrality tests [25].

Statistical tests for selective neutrality are often grouped into two classes based on the kind of data they employ: polymorphism-based tests and divergence-based tests. Polymorphism-based tests use intraspecific genetic variation to detect ongoing or relatively recent episodes of selection, which can be seen as deviations from neutral expectations in the site frequency spectrum (SFS) (e.g. Tajima's *D* [26] and Fay and Wu's *H* [27]) or the haplotype frequency spectrum (e.g. Ewens-Watterson *EW* test [28]). Such deviations are not, however, definitive evidence for selection since a complex demographic history can produce similar patterns [29], [30]. For example, an excess of high frequency alleles could be the result of either positive selection or a recent population bottleneck event. In contrast, an excess of low frequency alleles can be indicative of either the gradual accumulation of new mutations after a selective sweep event has carried beneficial (and linked) alleles to fixation or rapid population growth. One approach for reducing the influence of confounding factors, such as demography, background selection, and recombination, is to combine multiple neutrality tests that have different sensitivities to these factors into a compound test [18], [31], [32]. The *DHEW* compound neutrality test in particular has been shown through extensive simulation studies to have high power and specificity for detecting positive selection and it is relatively insensitive to recombination, background selection, population expansions and bottlenecks [32]. These features come from combining Tajima's *D* and Fay and Wu's *H*, which are highly sensitive to recombination, with the Ewens-Watterson *EW* test, which is largely unaffected by recombination [31], [32]. Additionally, Tajima's *D* is sensitive to background selection, population expansions and bottlenecks, while Fay and Wu's *H* is robust to these factors [18], [31].

The second major class of statistical neutrality tests is comprised of divergence-based tests that use interspecific comparisons alone or in combination with data on intraspecific polymorphism. By analyzing patterns of genetic divergence between species, these tests can detect selection long after it has occurred. Methods that combine polymorphism and divergence data, such as the McDonald-Kreitman (MK) test [33] and the Hudson, Kreitman, and Aguadé (HKA) test [6], [34], have increased power for detecting positive selection over other neutrality tests [35]. They are also relatively robust to recombination [6], [33], although, as with SFS tests, they can be sensitive to demographic history [36], [37].

While the variety of different tests for selective neutrality might appear excessive, some are more appropriate for particular forms of selection than others. For example, tests based on the ratio of nonsynonymous to synonymous sites (dN/dS) have proven to be very useful for detecting selection on amino acid residues that determine binding specificities of proteins that interact with rapidly evolving targets. Well-studied examples include gamete-recognition proteins of free-spawning marine invertebrates [38], [39], proteins involved in immune responses [40], pathogen-recognition proteins in plants [41], and viral epitopes that facilitate escape from the host's immune response and enhance transmission [42], [43]. In these examples, dN/dS ratios are very high because selection drives multiple amino acid replacements within binding sites. In contrast, selection acting on synonymous polymorphisms [44], [45], regulatory regions, or sites linked to genes [46] would go undetected by tests based on dN/dS. Similarly, the dN/dS ratio would not be much affected by a single amino acid replacement driven by selection. However, selection that drives the adaptive fixation of a single mutation can still be detected if it causes a selective sweep that reduces polymorphism in the chromosomal region linked to the selected site [47], [48].

For this study we used both polymorphism-based and divergence-based tests to detect positive selection on candidate genes in the blue crab, *Callinectes sapidus*, and its congener, *C. similis*, from which it is estimated to have diverged 13 My [49]. Both are common in estuarine and coastal waters from the United States mid-Atlantic coast to Colombia, with the range of *C*. *sapidus* extending further both north and south [50]. These species are similar in morphology, life history, development, and foraging behavior [51]–[53], but they are markedly different in their tolerances to hypoxia [54] and salinity extremes [55], [56]. As a result, *C*. *similis* is seldom found in salinities less than 15‰, whereas *C*. *sapidus* is commonly found in salinities ranging from 0 to 35 ‰ and can tolerate supersaline conditions (>35‰) [57]. Within coastal marsh and upper estuarine habitats, blue crabs (*C. sapidus*) often experience large, sudden changes in temperature, salinity, and dissolved oxygen. Environmental variation could select for adaptations to differing local conditions as well as tolerance to environmental variability itself. In contrast, these forms of selection have likely been weaker for *C. similis*, which encounters a much narrower range of physical conditions with less temporal variability.

We chose to investigate four candidate genes that we have recently shown undergo temporal changes in allele frequencies within a population of *C. sapidus* in the northern Gulf of Mexico as well as geographic differentiation on a broader scale [58]. Two of the candidate genes have putative roles in stress responses: the heat shock protein 70 (*hsp*) and trehalose 6-phosphate synthase (*tps*), while the other two candidate genes are involved in energy cycling and metabolism: ATP-synthase subunit 9 (*atps*) and ATP/ADP translocase (*ant*). For comparison, we included the ribosomal protein L12 (*rpl*) gene as a control since, as a housekeeping gene, it has no suspected role in environmental adaptation but is likely to be subject to purifying selection.

## Materials and Methods

### Ethics statement

The majority of the samples used in this study were collected in Louisiana, USA, on public lands and did not require permitting or permissions of any kind. Samples from Texas, USA, were collected by biologists from Texas Parks and Wildlife following state-approved sampling protocols. Non-lethal tissue samples were taken from crabs and live specimens were returned to their immediate environment whenever possible. Additional specimens from the Gulf of Mexico, Mexico, and Venezuela were generously provided by Dr. Darryl Felder from the University of Louisiana at Lafayette Zoological (ULLZ) Collection. No threatened or endangered species were sampled in this study.

### Sample collection

Adult, juvenile, and postlarval megalopae specimens of *Callinectes sapidus* were collected at several locations spanning approximately 300 km of the Louisiana Coast (Table 1) from May through October in 2010 and 2011. Crabs were collected using baited hand lines, hoop nets, and recreational vinyl-coated wire mesh traps. One walking leg was removed from each crab and stored in 95% ethanol prior to DNA extraction. Megalopae were collected using passive samplers, adapted from Metcalf et al. [59], which were deployed for either 24 h or for longer periods of up to two weeks. Collectors were rinsed in freshwater and megalopae were transferred from the rinse water to 95% ethanol. Whenever possible, all samples were stored at 4°C. Additional specimens of *C. sapidus* were collected in Lower Laguna Madre, Texas, during the summer of 2010 and stored in 95% ethanol prior to DNA extraction (Table 1). Archived specimens of *C. sapidus* from the University of Louisiana at Lafayette Zoological (ULLZ) collection that were collected in Mexico and Venezuela from 1999–2003 were also included in this study to increase the sampling range (Table 1). For interspecies comparison, three ULLZ specimens of *C. similis* that were collected on research cruises off the coast of Louisiana in June and July of 2010 were included.

**Table 1:** Sampling locations and life stage totals for *Callinectes sapidus*.

Country	State	Latitude (degrees)	Longitude (degrees)	Adult	Juveniles	Unknown	Megalopae	Totals
Mexico	Veracruz	*18.5302*	*−95.0262*	0	0	3	0	3
USA	Louisiana	29.8382	*−*93.3206	23	0	0	0	23
		29.5520	*−*92.3055	47	0	0	24	71
		29.2394	*−*90.0020	47	0	0	25	72
		29.6202	*−*92.1163	24	0	0	0	24
		30.3617	*−*90.1664	24	0	0	0	24
		29.2540	*−*90.6639	47	0	0	24	71
		29.5777	*−*91.8842	24	0	0	0	24
		29.7121	*−*92.7656	33	8	0	25	66
		29.7789	*−*93.1326	20	1	0	0	21
	Texas	26.2336	*−*97.1983	4	11	0	0	15
Venezuela	Falcon	*11.4593*	*−69.5778*	0	1	1	0	2
	Zulia	*10.9368*	*−72.0500*	2	1	2	0	5
		*10.9444*	*−71.4944*	0	1	0	0	1
		*10.4500*	*−71.6500*	0	0	2	0	2
		TOTALS		295	23	8	98	424

Italics indicate approximate sampling coordinates based on the location name associated with the cataloged specimen from the University of Louisiana at Lafayette Zoological (ULLZ) Collection (exact coordinates were not available).

### DNA extraction, PCR, and sequencing

Genomic DNA was extracted using either the PUREGENE DNA Purification Kit protocol for DNA isolation from marine invertebrate tissue (Gentra Systems, Inc.) or NucleoSpin 96 Tissue kits (Macherey-Nagel) on an automated liquid handling workstation (epMotion 5075 TMX, Eppendorf). DNA was extracted from entire megalopae or approximately 20 mg of leg muscle from adults and juveniles.

Primer pairs were designed to amplify between 191 and 600 bp of each gene using the polymerase chain reaction (PCR). Each PCR reaction included: 1.5 µl (10X) AmpliTaq Gold PCR buffer (Applied Biosystems), 1.5 µl (25 mM) MgCl_2_, 1.2 µl (10 mM) dNTPs, 0.9 µl (20 µM) of each forward and reverse primer, 0.6 units of AmpliTaq Gold (Applied Biosystems) and 5-25 ng of template DNA. All reactions were run on a Bio-Rad iCycler using the following profile: 10 min at 94°C; followed by 35 cycles of 20 s at 94°C, 20 s at 51.4–68°C, 30 s at 70°C; 5 min at 70°C; and held at 4°C. Primer-specific annealing temperatures and sequences are reported in Yednock and Neigel [58]. A portion of the mitochondrial 16S ribosomal DNA gene was also amplified from the megalopae using primers 16sar (5′-CGCCTGTTTATCAAAAACAT-3′) and 16sbr (5′-CCGGTCTGAACTCAGATCACGT-3′) from Palumbi et al. [60] to be sequenced for species identification using the following thermocycler profile: 10 min at 95°C; followed by 40 cycles of 1 min at 95°C, 1 min at 55°C, 1 min at 72°C; 2 min at 72°C; and held at 4°C.

Prior to sequencing, 3 µl of each PCR reaction was electrophoresed in an agarose gel stained with ethidium bromide to confirm amplification of a single product. The remaining PCR product was treated with 0.1 µl (20 U/µl) Exonuclease I (New England Biolabs, Inc.), 0.3 µl (5 U/µl) Antarctic Phosphatase (New England Biolabs, Inc.), and 6.6 µl milli-Q filtered water, then heated to 37°C for 1 h 15 min, 95°C for 5 min, and held at 4°C. Cycle sequencing reactions were performed in 10 µl total volume reactions with 4.5 µl milli-Q filtered water, 2.5 µl (5X) sequencing buffer [0.4 M Tris-HCl pH 9, 10 µM MgCl_2_], 2 µl (0.8 µM) primer, and 0.5 µl BigDye Terminator v.1.1 (Applied Biosystems). The thermocycling protocol followed Platt et al. [61]. Cycle sequencing products were cleaned by standard ethanol precipitation, rehydrated in 20 µl HiDi Formamide (Applied Biosystems), and denatured at 95°C for 3 min, then held at 4°C. All sequencing reactions were run on an ABI 310 Genetic Analyzer (Applied Biosystems) and basecalls were made with Sequencing Analysis software version 5.2 (Applied Biosystems) using the KB basecaller.

Sequences were aligned and edited in the SeqMan module of DNASTAR Lasergene software version 8.0.2 (DNASTAR, Inc.). End regions of poor quality were trimmed from sequences leaving 191–489 bp per locus for data analysis. With the exception of *atps* that included a portion of the 3′ UTR, all loci consisted exclusively of coding sequence. The haplotypic phase of edited sequences was determined using a Bayesian method implemented in PHASE v.2.1 [62], [63]. This program has been shown to be an accurate and robust method for reconstructing haplotypes from population genetic data [64], [65]. Unique sequences for *ant*, *atps*, *tps*, and *hsp* were deposited on GenBank (Accession numbers: KC886426 - KC886589). The sequences for *rpl* are shorter than the length requirement for submission to GenBank, therefore they were submitted to EMBL-Bank (HG530328 - HG530352).

### Sequence data analysis

To summarize variation across all sequences obtained from *C. sapidus*, standard descriptive sequence statistics were calculated for each gene region in DnaSP Version 5.10.01 [66]. These included the numbers of polymorphic sites (*S*) and haplotypes (*H*), haplotype diversity (*H*_d_), nucleotide diversity (*π*), and 4*Nµ* (*θ_W_*) estimated by Watterson's method [67], [68]. In addition, mean pairwise divergence estimated with the Jukes-Cantor correction for multiple substitutions (*K_JC_*) [68] was calculated from all *C. sapidus* and *C. similis* sequence pairs.

The minimum number of recombination events (*R_M_*) based on the four-gamete test [69] and the population recombination parameter γ (*4N_e_c*) [70] were estimated with the program SITES [70] for each locus. Sites with more than two possible alleles were excluded from *γ* and *R_M_* estimates for consistency with the assumed infinite sites model. The ZZ test [71] in DnaSP [66] was also used to assess the significance of intralocus recombination using 10^3^ coalescent simulations of neutral models assuming infinite sites and observed values of *θ_W_*. Mean linkage disequilibrium (LD) between pairs of sites within and between loci using the squared allelic correlation coefficient (*r* ^2^) was calculated in SITES and its significance was assessed by comparing observed values of *r* ^2^ with the distribution of simulated values from 1200 randomizations of the data.

### Neutrality tests

Polymorphism-based neutrality tests were applied to each locus separately using only *C. sapidus* sequences. Divergence-based neutrality tests were applied to data from *C. sapidus* and *C. similis*. Because combining coding and non-coding data can affect the results of some divergence-based tests [72], the 3′UTR (100 bases) of *atps* was excluded from these analyses.

For the polymorphism-based neutrality tests, departures from neutral expectations were detected with Tajima's *D* [26], Fay and Wu's normalized *H* [27], and the Ewens-Watterson *EW* homozygosity test [28]. Significance of all tests was determined in DnaSAM [73] using 10^3^ simulations that assumed a neutral model and no recombination. Because *D*, *H*, and *EW* differ in their sensitivities to demographic history, background selection, and recombination, the *DHEW* compound test [32] was also performed using the Perl script provided with DnaSAM [73]. The multidimensional rejection region of the *DHEW* test was determined for each locus from 10^4^ coalescent simulations conditional on the locus-specific *θ_W_* and a Bonferroni-corrected [74] nominal threshold of P = 0.01.

The HKA test [34] was used to determine if the relationship between intraspecific polymorphism and interspecific divergence was consistent across loci, as is expected for neutral loci that differ only in mutation rate. The HKA test was implemented with the program HKA provided by Jody Hey (http://genfaculty.rutgers.edu/hey/software). Because the number of sequences in the complete data set exceeded the sample size limit of the program, we performed HKA tests on 100 randomized subsamples of the data set. Each subsample consisted of both sequences from each of 172 randomly selected individuals of *C. sapidus* and all available sequences (*n* = 6) of *C. similis*. This re-sampling method allowed analysis of the complete data set and provided a way to assess the sensitivity of the results to data sampling.

The maximum likelihood HKA test (MLHKA) developed by Wright and Charlesworth [6] was used to extend the results of the HKA test by specifically testing the neutrality of *ant*, *atps*, *tps*, and *hsp*. MLHKA uses a likelihood ratio test to compare a model of neutral evolution for all loci with models in which one or more loci are assumed to be under selection. The MLHKA test also uses the level of diversity at a locus to estimate the selection parameter *k*, which reflects the relative strength and type of selection (i.e. balancing selection or a selective sweep) acting on a locus. The MLHKA test was run for all of the subsampled data sets used in the HKA analysis. Each MLHKA run included 10^5^ MCMC chains, and statistical significance was evaluated by comparing twice the difference of the log-likelihood scores between the neutral model and the selection model to a χ^2^ distribution. Degrees of freedom were defined by the difference in the number of parameters between the two models being compared. The MLHKA test was used to evaluate two sets of candidate loci: (1) the complete candidate locus model in which *ant*, *atps*, *tps*, and *hsp* were considered to be under selection and *rpl* was assumed to not be under selection, and (2) a condensed candidate model in which only loci showing exceptionally high or low ratios of polymorphism to divergence (θ_π_/*K_JC_*) were considered as candidate loci.

The MK test [33] was used to examine the relationship between intraspecific polymorphism and interspecific divergence for synonymous and nonsynonymous (amino acid altering) nucleotide substitutions. Under a neutral model, the ratio of nonsynonymous to synonymous substitutions is expected to be the same for within species polymorphism (P_N_/P_S_) and between species divergence (D_N_/D_S_). In contrast, adaptive fixations would cause D_N_/D_S_ to be greater than P_N_/P_S_. The MK test was performed in DnaSP [66] for each locus using all available *C. sapidus* (n = 848) and *C. similis* (n = 6) sequences.

### Historical demography

**Figure 1:**
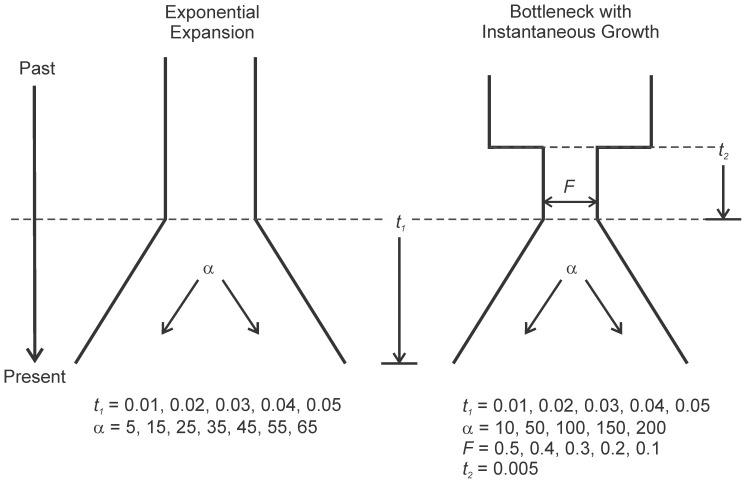
Demographic models evaluated in simulations. Demographic models and parameter values that were evaluated in coalescent simulations. *t*_1_, time of expansion; *t*_2_, length of bottleneck event; α, population growth parameter; *F*, bottleneck size defined as a proportion of the present day population (*N*_e_).

Coalescent simulations of exponential population expansion and bottleneck models (Figure 1) were performed for a range of parameter values using the program ms [75], as implemented in DnaSAM [73]. The expansion models simulated a population increasing in size (such as from a refugium), while the bottleneck models included an abrupt reduction in population size (with loss of genetic variation) prior to the expansion event. Values for time parameters were chosen to represent the historical environmental and geological events that were most likely to impact the demography of *C. sapidus* (see Discussion). All models included a parameter for the time elapsed from the start of population expansion to the present (*t*_1_) with values ranging from 0.01 to 0.05, which correspond to approximately 10,000 to 53,000 years. All models also included a parameter for the rate of population growth (α) during the expansion; values for the expansion model ranged from 5 to 65 and for the bottleneck model from 10 to 200. Bottleneck models also included a population bottleneck size parameter (*F*), defined as a proportion of *N*_e_ (ranging from 0.1–0.5), and a time of reduction (*t*_2_) corresponding to 5,000 years prior to expansion (Figure 1). Simulations were run separately for each locus based on the observed locus-specific *θ_W_* (Table 2) and included 10^3^ replicates for each parameter combination. In total, 35 parameter combinations were evaluated for the population expansion model and 125 for the bottleneck model. Tajima's *D*, Fay and Wu's *H*, and the Ewens-Watterson *EW* test were calculated from each simulation to generate distributions for significance testing. Observed values for *D* and *H* were considered significantly different than those generated from the demographic model if they fell in the lower 1% tail of the simulated distribution, based on a Bonferroni-correction [74] for multiple loci. *EW* was considered significantly different if it fell in the upper 1% tail of the simulated distribution.

**Table 2:** Sequence information and summary statistics for *Callinectes sapidus*.

Locus	Length	%	*S*	*NS*	*Syn*	*H*	*H*_d_	*π*	*θ_W_*	*θ_π_*	*K_JC_*	*θ_π_/K_JC_*
*ant*	414	40	33^a^	2	32	54	0.757	0.003	4.509	0.011	0.019	0.567
*atps*	227	46	27	0	27	39	0.662	0.005	3.689	0.009	0.003	3.215
*tps*	368	13	22	5	17	19	0.313	0.001	3.006	0.008	0.054	0.152
*hsp*	489	23	28	12	16	31	0.757	0.003	3.825	0.008	0.019	0.405
*rpl*	191	36	11	1	10	23	0.819	0.009	1.503	0.008	0.014	0.574

*Length*, trimmed length of analyzed sequence; *%*, percent coverage of the complete mRNA reference sequence available on GenBank (*ant*: EF077712, *tps*: EU679406, *hsp*: DQ663760, *rpl*: FJ774832) and the partial mRNA of *atps* (HM217802); *S*, total number of segregating sites; *NS*, number of nonsynonymous segregating sites; *Syn*, number of synonymous segregating sites; *H*; number of haplotypes; *Hd*, haplotype diversity; *π*, nucleotide diversity; *θ_W_*, Watterson's theta; *θ_π_*, theta pi, *K_JC_*, mean divergence between *C. sapidus* and *C. similis*, defined as the mean number of nucleotide differences per site between species.

a Indicates one haplotype has a *Syn* and *NS* allele, therefore the value of *S* is 1 less than the sum of *NS* and *Syn*.

## Results

### Sequence data analysis

Among the five loci, 122 nucleotide substitutions were found at 121 polymorphic sites in *C. sapidus*; of these 102 were synonymous and 20 were nonsynonymous. Haplotype diversity (*H*_d_) ranged from 0.313 (*tps*) to 0.819 (*rpl*) (Table 2). On average, coding sequences had one polymorphism every 15.9 bases, while the non-coding 3′ UTR region of *atps* had one polymorphism every 5.4 bases. Mean divergence (*K_JC_*) between *C. sapidus* and *C. similis* was highest for *tps* (0.054) and lowest for *atps* (0.003) (Table 2). Nucleotide diversity (π) and Watterson's theta (*θ_W_*) also varied across loci (Table 2).

**Figure 2:**
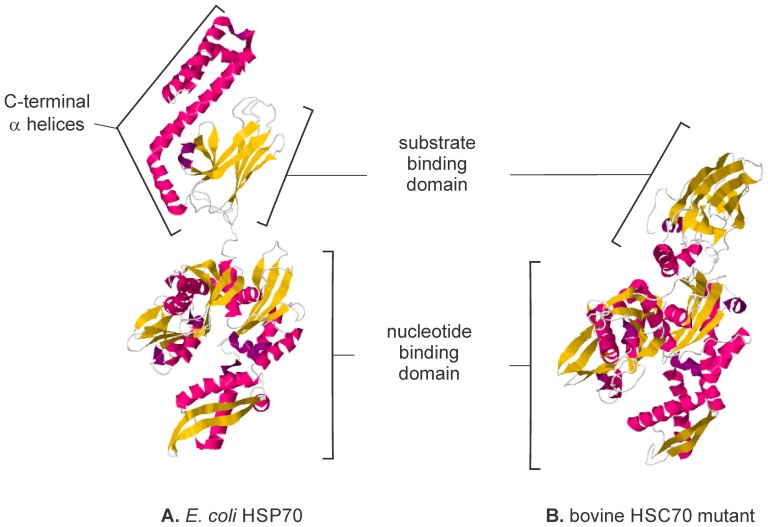
Comparison of complete and truncated isoforms of the 70 kDa heat shock protein family. (A) Protein structure of *E.coli* HSP70 (DNAK) chaperone (aa 1–605) complexed with ADP and substrate (PDB: 2KHO). (B) The truncated bovine HSC70 (aa 1–554) mutant E214A/D214B in post-ATP bound phase (PDB: 1YUW). The truncated form is missing a portion of the C-terminal α-helices, but retains the ATPase site within the nucleotide binding domain.

Six specimens of *C. sapidus* were heterozygous for a nonsense substitution within the region of *hsp* corresponding to the HSP70 C-terminal domain. Resequencing confirmed that these sequences were correct and not the result of base calling errors or sequencing artifacts. Thorough examination of the chromatogram sequence traces for *hsp* (n = 854) as well as the other four loci provided no evidence for paralogous loci or pseudogenes in the form of triple peaks. The C-terminal position of the nonsense mutation in *hsp* would terminate the protein sequence 97 amino acid residues early, but not before the protein's essential substrate-binding or ATPase sites (Figure 2). Therefore, the truncated version of the HSP70 protein that is presumably translated from this mutant allele could still be functional (see Discussion).

Intralocus linkage disequilibrium (LD) was significant for all loci except *ant*, based on the randomized *r^2^* test. The *ant* locus also had the highest estimates for the minimum number of recombination events (*R_M_*) and the population recombination parameter γ (*4N_e_c*) (Table 3). However, no locus showed significant evidence of intralocus recombination based on the ZZ simulation test (Table 3), therefore intralocus recombination is not expected to affect the results of the neutrality tests. Interlocus LD was significant only for an association between *hsp* and *rpl* (*r^2^* = 0.0013, *p*<0.025), but this was not significant following a Bonferroni correction (α = 0.005) [74].

**Table 3:** Linkage disequilibrium (LD) and recombination results for *Callinectes sapidus*.

Locus	*r^2^*	*γ*	*R_M_*	*γ/bp*	*ZZ_O_*	*p(ZZ)*
*ant*	0.002	9.829	4	0.024	0.001	0.643
*atps*	0.006*	0.000	3	0.000	−0.006	0.401
*tps*	0.060*	0.000	0	0.000	0.071	0.897
*hsp*	0.011*	0.000	2	0.000	−0.009	0.438
*rpl*	0.030*	1.160	2	0.006	0.026	0.757

*r^2^*, mean pairwise LD; *γ*, population recombination rate; *R_M_*, minimum number of recombination events; *γ/bp*, population recombination rate per base pair; *ZZ_O_*, observed ZZ; *p(ZZ)*, probability of simulated ZZ being as extreme or more extreme than *ZZ_O_*.

*indicates *p*<0.005.

### Neutrality tests

All neutrality tests with *C. sapidus* sequences were performed on three different sample partitions of sequences to assess whether the results were sensitive to the geographic origins of the sequenced individuals. These partitions consisted of sequences from: 1) all locations combined, 2) US locations only, and 3) Louisiana locations only. Results were consistent across all three partitions, indicating no bias from potential population structure, therefore only the results for the full data set are reported.

Departures from neutral expectations were detected for *tps* as significant Tajima's *D*, Fay and Wu's *H*, and Ewens-Watterson *EW* tests (Table 4). Together these results reflect an excess of rare alleles (negative *D*) and high-frequency alleles (positive *H*), and levels of homozygosity (*EW*) for *tps* that can be interpreted as evidence for a selective sweep. These results are further supported by a significant *DHEW* test for *tps* (Table 4), which, relative to all of the individual tests, provides more power for detecting a selective sweep resulting from positive selection and is more robust to demography and recombination [32]. Significantly negative values of *D* were also found for *ant* and *atps*, but the *H*, *EW*, and the compound *DHEW* tests were not significant for these loci (Table 4).

**Table 4:** Tajima's *D*, Fay and Wu's *H*, Ewens-Watterson *EW*, and *DHEW* compound test results for *Callinectes sapidus*.

Locus	*D*	*H*	*EW*	*DHEW(P)*
*ant*	−2.055*	−0.753	0.424	0.102
*atps*	−1.756*	−0.436	0.398	0.103
*tps*	−2.011**	−6.337**	0.723*	0.105*
*hsp*	−1.622	0.269	0.244	0.104
*rpl*	0.590	−1.377	0.192	0.125

*D*, Tajima's *D*; *H*, Fay and Wu's *H*; *EW*, Ewens-Watterson *EW*; *DHEW(P)*, critical *P* for *DHEW* test. Significance of the *DHEW* test at nominal α = 0.01 is based on the p-values for *D* and *H* being less than *DHEW(P)* and the p-value of *EW* being greater than 1-*DHEW(P)*. Significance of the individual *EW* test is based on 1 minus the p-value of *EW*.

*p<0.01, **p<0.001.

Significant (α = 0.05) departures from neutral expectations were detected in a series of HKA tests that evaluated the consistency of intraspecific polymorphism and interspecific divergence across loci. This analysis included 100 random subsamples of the complete data set and nearly all (98%) showed significant variation across loci (mean *p*-value  = 0.018). For 18% of the same 100 subsampled data sets, the MLHKA test showed significant differences between a null model that assumes neutrality for all loci and the complete candidate model in which *ant*, *atps*, *tps*, and *hsp* were all assumed to be under selection. The divergence to polymorphism ratios (θ_π_/*K_JC_*) for *atps* (3.215) and *tps* (0.152) differed considerably from those of the remaining three loci (Table 2); therefore *atps* and *tps* were included in the condensed candidate model. This condensed model significantly improved the likelihood compared to the neutral model for 99% of the subsamples. The selection parameter *k*, as defined by Wright and Charlesworth [6] and estimated by the MLHKA test, reflects the degree to which diversity at a locus is increased (*k*>1) or decreased (*k*<1) relative to expected diversity under a neutral model. The value of *k* for *atps* ranged from 5.97 to 56.20 (mean  = 16.73) across all data subsamples. These values reflect higher levels of intraspecific polymorphism relative to interspecific divergence and are consistent with expectations of balancing selection. In contrast, the range of *k* for *tps* was much smaller and consistently less than one (0.16–0.42; mean  = 0.30), which is indicative of reduced diversity resulting from a selective sweep.

The MK test for *ant* showed a significant departure from neutral expectations (Table 5); the ratio of nonsynonymous to synonymous mutations fixed between species (D_N_/D_S_  = 1.5) was significantly greater than the within-species polymorphism ratio (P_N_/P_S_  = 0.06). No significant departure from neutrality was found for *tps* with the MK test (Table 5). MK tests could not be conducted for *atps*, *hsp*, or *rpl* because there were no fixed differences between *C. sapidus* and *C. similis* at these loci.

**Table 5:** McDonald-Kreitman test results for *ant* and *tps.*

Locus	Mutation Class	Fixed	Polymorphic	Fisher's Exact (p)
*ant*	*Nonsynonymous*	3	2	
	*Synonymous*	2	34	0.0086*
*tps*	*Nonsynonymous*	2	4	
	*Synonymous*	10	19	1.0000

McDonald-Kreitman tests for *ant* and *tps* using *C. sapidus* polymorphisms and fixed differences between *C. sapidus* and *C. similis*. Contingency tables for *atps*, *hsp*, and *rpl* could not be calculated because there were no fixed differences between *C. sapidus* and *C. similis*.

*p<0.025.

### Historical demography

**Figure 3:**
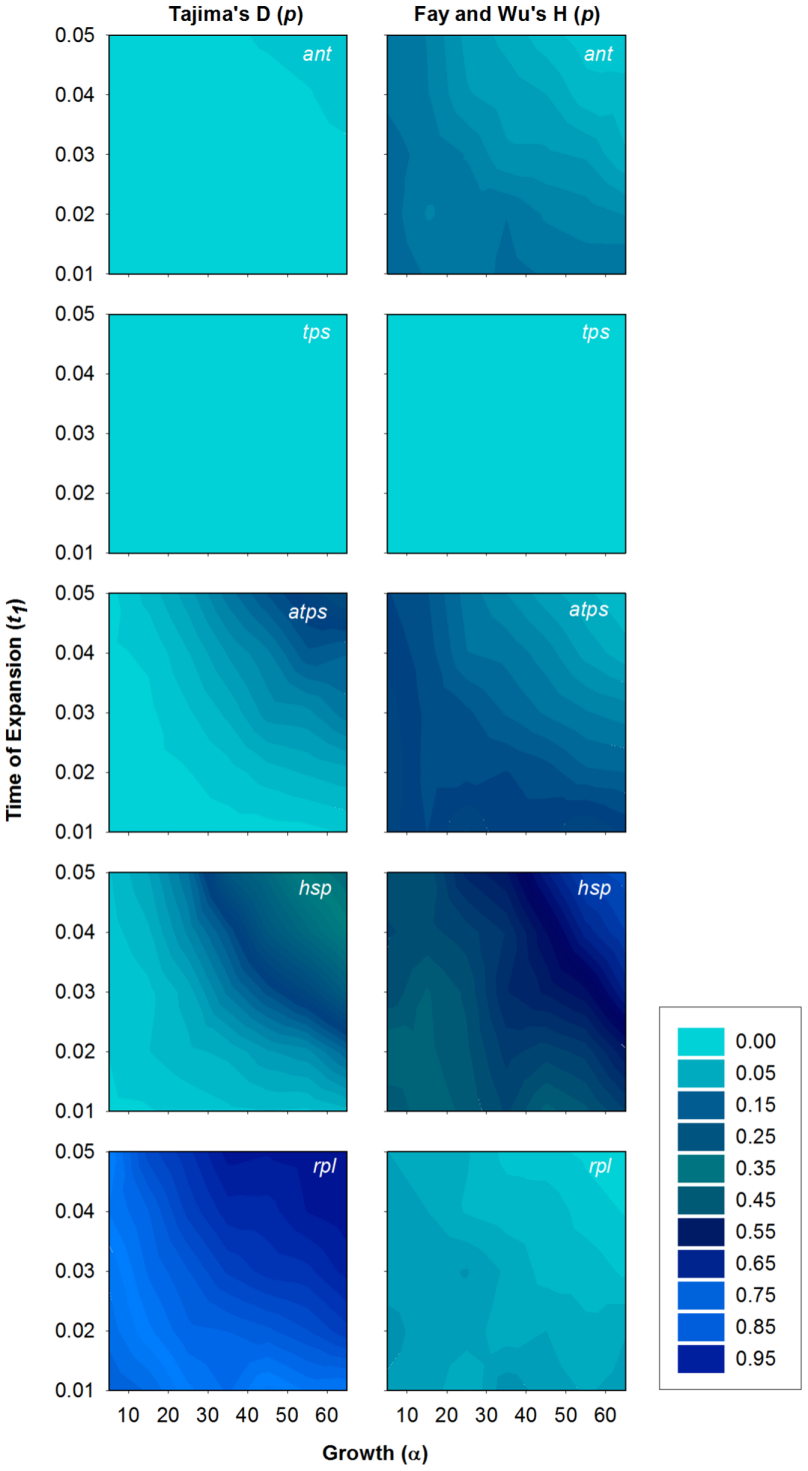
Results of population expansion simulations. P-values for Tajima's *D* and Fay and Wu's *H* tests from coalescent simulations of population expansion scenarios across a range of growth (α) and time of expansion (*t*_1_) parameter values. The demographic model is shown in Figure 1.

**Figure 4:**
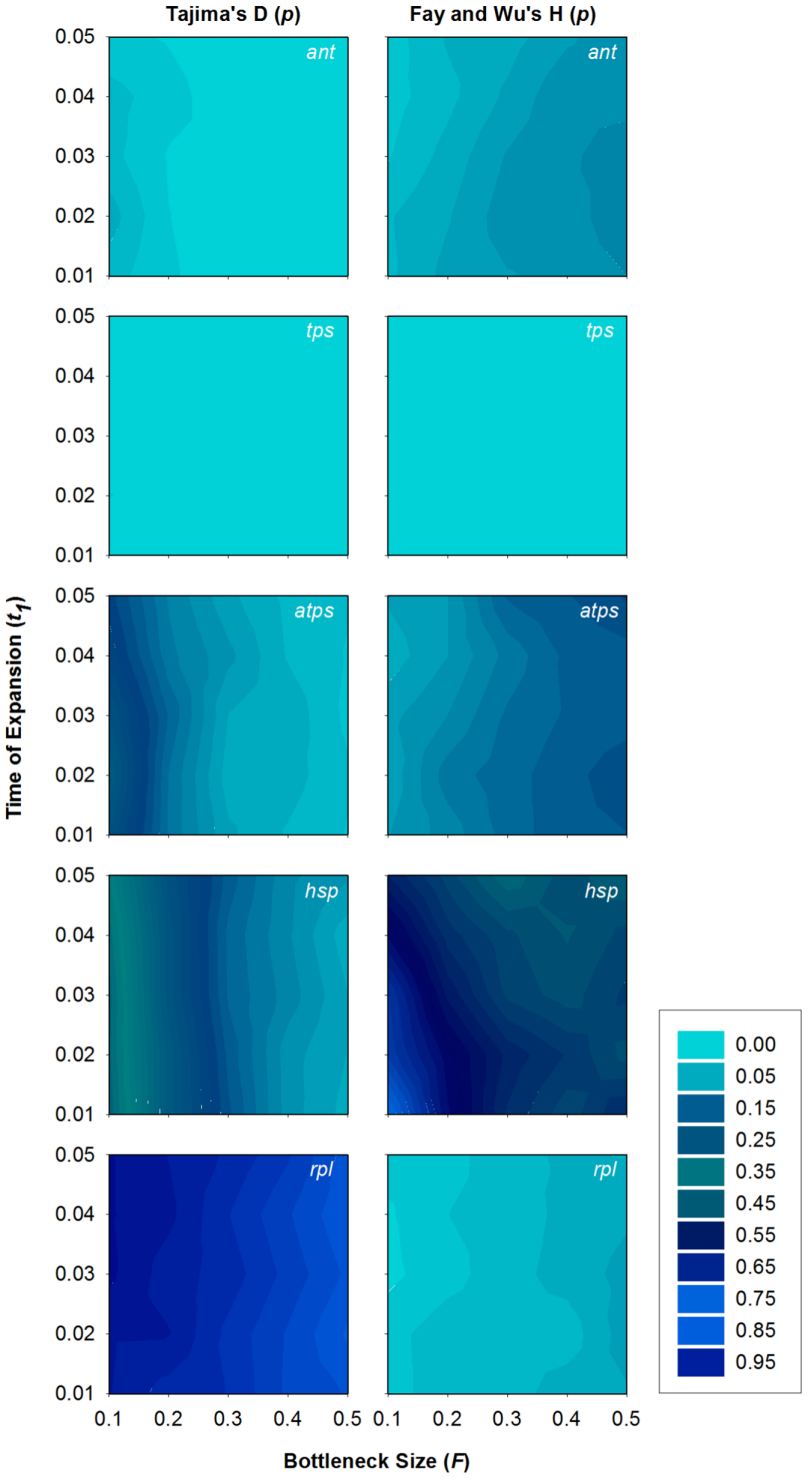
Results of population bottleneck simulations. P-values for Tajima's *D* and Fay and Wu's *H* tests from coalesceant simulations of bottleneck scenarios run with a constant growth rate (α = 10) since the initial time of expansion (*t*_1_). Bottleneck size (*F*) is defined as a proportion of the present day population (*N*_e_). The results of simulations with α = 50, 100, 150, and 200 show similar p-values for each locus. The demographic model is shown in Figure 1.

Several parameter combinations in the coalescent simulations of neutral population expansions and bottlenecks produced distributions of *D* and *H* that were not significantly different (*p*>0.01) than the observed values for *atps*, *hsp*, and *rpl* (Figures 3,4). Similarly, for *ant* most of the simulation models could also explain the observed value for *H*, but only a small number of parameter combinations were consistent with the observed value for *D* (Figures 3,4). In contrast, the observed value of *H* for *tps* could not be explained by any of the simulated demographic models (Figures 3,4) and, except for four bottleneck scenarios (out of 125), *D* for all of the demographic scenarios differed significantly from the observed value for *tps* with a Bonferroni-corrected α = 0.01. For those four bottleneck scenarios with values of *D* that did not differ significantly, the observed value of *D* was always found within the lower 1.7% tail of their distributions, indicating very little overlap between the observed and simulated values. For all loci, the simulated distributions of *EW* from all expansion and bottleneck models were not significantly different than the observed values, with the exception of two expansion scenarios for *tps*.

## Discussion

Several lines of evidence from this study suggest *tps*, or a closely linked locus, has undergone positive selection in *C*. *sapidus*. Signatures of positive selection were found at *tps* by all polymorphism-based tests and the divergence-based MLHKA test. While these tests should generally be interpreted with caution since demographic history and selection can produce similar patterns, in this study there are several lines of evidence that support the interpretation of a selective sweep affecting *tps*.

First, because all diploid loci are subject to the same population history, demographic factors creating the false appearance of selection on one locus would be expected to have similar effects on other loci. This was not seen here; all polymorphism-based neutrality tests were significant for *tps*, but not for the other four loci. Additionally, the compound *DHEW* test was significant for *tps*, but not for the other four loci. Unlike the individual polymorphism-based tests, the compound *DHEW* test is relatively insensitive to demography, background selection, and recombination and it has higher power for detecting positive selection than the individual tests [32]. This stems from the test's high specificity for detecting selective sweeps that result from positive selection, which is gained from combining tests that are sensitive to different confounding effects in mutually exclusive ways [32]. Second, the MLHKA analysis provided highest support for a model in which *tps* and *atps* were the only loci assumed to be under selection, with the estimates for the selection parameter *k* suggesting *tps* experienced positive selection. Finally, the potential influences of demography on the polymorphism-based test statistics was directly evaluated with coalescent simulations.

While the demographic history of *C. sapidus* is unknown, repeated glaciation events of the Pleistocene caused significant changes in sea level [76] and available marsh habitat for *C. sapidus* in the Gulf of Mexico (GOM) and elsewhere in its range. Winter surface water temperatures along the continental margins of the GOM are thought to have been 4–5°C cooler approximately 18,000 years ago, which would have displaced the 20°C winter isotherm to the southernmost regions of the GOM [77]. During this time the ranges of temperate and tropical intertidal species would also have been pushed to lower latitudes [78] until the end of the last glacial period approximately 12,000 years ago when expansion northward would have begun. A wide range of population expansion and bottleneck scenarios corresponding to the estimated timing of these events was evaluated in this study and none of the demographic models could explain the observed value of *H* for *tps.* Similarly, except for four out of 125 bottleneck scenarios, none of the demographic models could explain the observed value of *D* for *tps* either. The four bottleneck scenarios that could explain *D* included a severe population reduction to 10–20% of the current population size approximately 20,000–40,000 years ago, followed by very slow population growth. While these bottleneck scenarios can produce the observed value of *D*, they did so only rarely, with a maximum probability of 0.017 for any single simulation run. It should be noted that the demographic models evaluated in this study were not exhaustive, but the broad range of demographic parameter values that were explored was chosen to represent the most plausible histories for *C*. *sapidus* based on available information for the geologic history of the GOM.

In contrast to the values of *D* and *H* for the *tps* locus, values for *EW* were consistent with several of the expansion and bottleneck scenarios that we tested. To interpret these results it is important to recognize that the *EW* test is based only on the spectrum of allele (haplotype) frequencies, while the *D* and *H* tests are based on the frequencies of individual segregating sites. Furthermore, a simulation study [35] indicated that, relative to Tajima's *D*, the power of the *EW* test to detect a selective sweep decreases as the advantageous allele approaches fixation and as *θ* (4*Nµ*) increases.

The production and physiological roles of trehalose have been well studied in invertebrates and provide insight into why *tps* might be a target for selection [79]–[81]. The *tps* gene encodes two functional enzyme domains involved in trehalose biosynthesis: trehalose 6-phosphate synthase (TPS), which catalyzes the production of trehalose 6-phosphate by transferring glucose from UDP-glucose to glucose 6-phosphate; and trehalose 6-phosphate phosphatase (TPP), which converts trehalose 6-phosphate to trehalose. Trehalose is a disaccharide composed of two glucose molecules connected by an α,α-1,1-glycosidic linkage. This structure makes trehalose an unusually stable sugar, and in many species it helps to maintain cell and protein integrity during exposure to environmental stress. For example, increased trehalose production has been linked to desiccation, dehydration, oxidation, anoxia, and hypoxia [79], [80]. Trehalose also acts as a general protein stabilizer during temperature stress by increasing temperatures at which proteins denature [82]. In crustaceans, trehalose production has been linked to changes in salinity [83], pathogen response [84], pathogen-simulated lipopolysaccharide challenge [85], and extreme temperatures [86]. Any of these stressors could be responsible for the signature of positive selection found at *tps*. The coastal environment in which *C. sapidus* is found experiences drastic shifts in temperature, salinity, and dissolved oxygen. Changes in temperature and salinity could also influence exposure to pathogens [87], [88]. In addition, the planktonic larval stage of this species encounters a dynamic suite of environmental stressors during development in open waters. We suggest that *tps* be considered a likely candidate for selection, but further assessment should include investigation of possible functional differences among the gene products of different alleles for the entire coding sequence as well as linked regulatory regions.

The neutrality tests implemented in this study detect different kinds of deviation from neutral expectations that can have different underlying causes. For example, none of the polymorphism-based tests were significant for *atps*, with the exception of Tajima's *D*, which could be explained by any number of demographic scenarios. However, the MLHKA test, which combines polymorphism data with divergence data and is more robust to demography, provided strong evidence that *atps* has undergone balancing selection. Additionally, the MK test was not significant for *tps*, despite the evidence for selection found from the other neutrality tests. It's important to note that the MK test differs from the others used here in that it compares synonymous and nonsynonymous substitutions within and between species. Selection acting on codons outside the region we examined, on a small number of amino acid replacements, or on linked sites would not be detected.

The MK test for *ant* was significant (Table 5) and indicates that selection may have favored amino acid substitutions in this gene region. The significance of this test is based on the ratio of nonsynonymous to synonymous mutations fixed between *C. sapidus* and *C*. *similis* being significantly higher than the ratio observed within *C. sapidus* sequences alone. There is evidence that significant MK tests can result from population expansion if some of the nonsynonymous substitutions are slightly deleterious [37], therefore a significant result cannot be interpreted as conclusive evidence for positive selection. However, further investigation into the functional effects (if any) of the observed amino acid replacements and other coding regions in *ant*, may provide insight into the adaptive evolution of this gene within the genus *Callinectes*.

There was no evidence of selection acting on *rpl*, which is consistent with our *a priori* designation of *rpl* as a control for which segregating variation is likely to be neutral. Based on the congruence in the results for *hsp* and *rpl*, it appears that variation at *hsp* is either neutral or under a form of selection that is not readily detected by the methods employed here. The stop codon found in one *hsp* allele also might suggest that *hsp* is not under strong selection. However, the functional consequences of this stop codon are unknown, so it is impossible to determine if it results in a functionally null allele. The presence of null alleles in protein-coding regions is not very unusual. Allozyme studies report frequencies of null alleles as high as 0.012 for *Drosophila melanogaster* [89] and 0.059 for *Pinus resinosa* [90]. The frequency of the *hsp* allele observed in the present study (0.007) is well under these previously reported frequencies. It is also possible that this allele encodes a truncated, fully functional HSP70 protein. Two of the 14 identified members of the HSP70 family in *Arabidopsis* encode HSP70 proteins that are truncated at their C-terminal ends, and one is known to be expressed [91]. Hundley et al. [92] found the truncated protein product from a mutant *hsp* gene in yeast to be fully functional. The *stch* gene also encodes a fully functional HSP70-like protein with a truncated C-terminal domain [93]. A comparison of the *hsp* allele found in this study with *stch* orthologs from humans, rats, and *C. elegans* [94] shows the *stch* gene is truncated 88 amino acids prior to the *hsp* allele found in this study and about 78 amino acids prior to the human HSP70 (data not shown).

## Conclusions

Targeted DNA sequencing of candidate genes provides a promising technique for investigating natural selection in non-model organisms. We examined variation within partial sequences of five nuclear protein-coding genes for a large sample of *C*. *sapidus* and found patterns consistent with selection acting on *tps* that could not be explained by any of the demographic scenarios that were evaluated in coalescent simulations. Evidence was also found for balancing selection at *atps* and positive selection at *ant*. These results suggest future studies on the functional capacities of *tps*, *atps*, and *ant* may elucidate the patterns observed in this study.
